# Family SES, family social capital, and general health in Chinese adults: exploring their relationships and the gender-based differences

**DOI:** 10.1186/s12889-020-09508-5

**Published:** 2020-09-14

**Authors:** Ying Ji, Qingping Yun, Xuewen Jiang, Chun Chang

**Affiliations:** School of Public Health, Peking University, No.38 Xueyuan Road, Haidian District, Beijing, 100191 China

**Keywords:** Family SES, Family social capital, General health, Gender-based difference

## Abstract

**Background:**

Numerous studies have clarified that family socioeconomic status (SES) is positively associated with health. However, the mechanism of family SES on health needs to be further investigated from a social epidemiological perspective. This study aims to analyze the relationships among family SES, family social capital, and adult general health and tests whether gender-based differences exist in the relationship between family social capital and general health.

**Methods:**

A cross-sectional survey was used to collect data from 4187 representative households in six Chinese provinces. Family SES was conceptualized based on household income, family education, and family occupational status. Family social capital was measured by using family cohesion and health-related family support. General health was assessed by using five general health perception items of the Health Survey Short Form. Structural equation modeling (SEM) was applied to examine the relationships among family SES, family social capital, and general health, and a linear regression model was used to test gender-based differences.

**Results:**

The SEM showed that the direct effects of family SES, family cohesion, and health-related family support on health were 0.08 (*P* < 0.001), 0.17 (*P* < 0.001), and 0.10 (*P* < 0.001), respectively. Family SES had indirect effect (β = 0.05, *P* < 0.01) on general health via health-related family support. The total effect of family social capital (β = 0.27, *P* < 0.001) on general health was greater than that of family SES (β = 0.13, *P* < 0.001). Besides, the regression showed that the effect of health-related family support on general health was greater for women (β = 0.13, *P* < 0.001) than men (β = 0.04, *P* > 0.05).

**Conclusions:**

The results provide strong support for the positive association between family SES, family social capital, and adult health. Family intervention programs should focus on establishing a harmonious family relationship to mobilize family support, particularly for the families with low cohesion and low SES.

## Background

Socioeconomic status (SES) is one of the fundamental factors determining an individual’s health status [1]. More and more studies have tried to understand the impact of family SES on health from different perspectives. From the perspective of family investment, good household economic conditions can shape a number of life circumstances—quality of housing, neighborhood conditions, and access to medical care—all of which carry significant health implications. Considering the health belief perspective, families with higher education level are more likely to have better health beliefs and healthy behaviors than families with lower education level [2, 3]. Considering the social epidemiology perspective, high family SES is also related to high family social capital [4], and family social capital is positively associated with children’s well-being [5]. It is worthwhile to investigate the potential mechanisms of these three components from the social epidemiology perspective.

Before investigating the relationships among family SES, family social capital, and health, it is necessary to first understand the various definitions of family social capital as well as the available measurements. The Dictionary of Epidemiology defines social capital in terms of resources falling into two categories: that is, resources (1) available to members of social groups (e.g., social trust and social cohesion) and (2) embedded within individuals’ social networks (e.g., social support) [6]. Coleman was one of the earliest scholars to introduce social capital into the family context. He defined family social capital as a form of capital that enables families to successfully manage the material and symbolic resources they hold for the benefit of their members [7]. Although different perspectives and dimensions have been used to measure family social capital in health-related literature, family cohesion and family support have consistently been the two main dimensions [8], which is consistent with the views of social cohesion and social network [9].

Family cohesion is the feeling of emotional closeness with family members [10], it is assessed using different scales in adult population [11–13]. Generally, family cohesion scales consist of a set of items, including family members’ respect for one another, tendency to get along with each other, shared values, and trust on each other. Previous studies show that family cohesion is positively associated with the health behavior of family members [14], and that greater family cohesion is associated with better health outcomes for children [15, 16].

Family support refers to the practical assistance, encouragement, and care offered by the family as received or perceived by an individual [17]. Family support (e.g., children presented, financial support) serves as an enabling factor and facilitates health care use among the older population [18]. Besides, family support plays an important role in stress resistance and disease rehabilitation [19, 20]. It was found that individuals with higher family support are more likely to report better health status [21].

There is a necessity to observe gender differences when investigating the relationship between social capital and health. In a society where gender plays a determining role in daily activities, men tend to rely on the social capital of workplaces while women tend to rely on the social capital of families [22]. Due to the differences in social networking between men and women, previous studies suggest that the impact of individual social capital on health may be unequal for men and women [23, 24]. Determining whether this gender difference still exists in the relationship between family social capital and health requires further research.

**Fig. 1:**
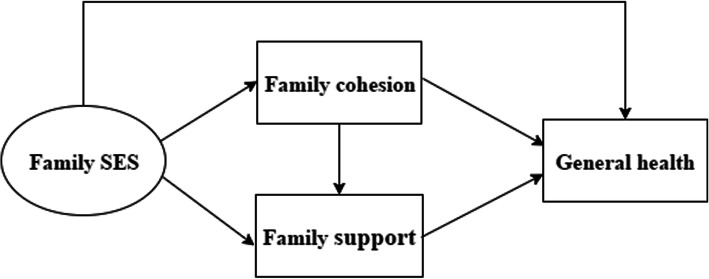
The conceptual framework

Guided by the existing evidence on the role of family SES and family social capital in influencing health, a conceptual framework is developed (Fig. 1). The primary aim of this study is to investigate the potential association between family SES, family social capital, and general health by using Chinese household data. Besides, gender differences in the relationship between family social capital and health are tested.

## Methods

### Study design and data source

The size of the study sample was estimated by using the following formula with four parameters: *n* = DF* *Z*_*α*_^2^ * S^2^/d^2^.

The four parameters are as follows:

1. Standard deviation (S): General health was chosen as the key indicator to compute sample size. According to the existing data on the Chinese population [25], S ranged from 19.9 to 20.4. We set S = 20.
2. Precision: We set the relative error at less than 5%. Hence, the absolute error was found to be 1.
3. Alpha level: We set α = 0.05 two-tailed, and hence *Z*_*α*_ = 1.96.
4. Design effect (DF): We set DF = 2, similar to most studies on this subject. Considering that the non-response rate was 10%, the sample size for this study is 3381.

A cross-sectional survey was used to collect data from the national sample of family households. Based on the level of economic development, 31 province-level regions, except Hong Kong, Macau, and Taiwan, were classified into eastern, central, and western regions. Administratively, four divisional levels—including province-, county-, township-, and committee-levels, were used in the sampling methods. Multistage random sampling methods were used, and the regions were selected from stages 1 to 4 by utilizing the random digits table. In stage 5, for each selected committee, 30 households were selected via systematic sampling based on the house number (Additional file Table 1). A total of 6 province-level regions, 24 county-level regions, 72 township-level regions, 144 committees, and 4320 family households were selected in our study. Only one family member was chosen from each selected family household for a face-to-face interview. The criteria for the subjects’ inclusion were 1) 18 years old or above and 2) familiarity with the family situation. The survey was conducted between June 20, 2014, and July 22, 2014. A total of 4187 households took the survey, and the response rate was 96.8%.

**Table 1:** Demographic characteristic of participants, and general health and family social capital score in different population groups

	Demographic characteristic	General health	Family cohesion	Health-related family support
	n (%)	Mean ± SD	Mean ± SD	Mean ± SD
Total		71.66 ± 20.92	74.77 ± 8.32	7.12 ± 2.59
Gender				
Male	1576 (37.6%)	73.60 ± 20.34	75.02 ± 8.13	6.85 ± 2.68
Female	2611 (62.4%)	70.49 ± 21.18	74.62 ± 8.43	7.23 ± 2.53
		*P* <0.001	*P* = 0.136	*P* <0.001
Age group				
18–30 years old	503 (12.0%)	78.49 ± 16.63	74.84 ± 8.43	6.88 ± 2.40
31–40 years old	752 (18.0%)	75.66 ± 19.40	76.07 ± 7.60	7.11 ± 2.47
41–50 years old	1397 (33.3%)	71.39 ± 21.34	74.63 ± 8.26	6.89 ± 2.61
51–60 years old	1005 (24.0%)	68.07 ± 22.07	74.39 ± 8.70	7.29 ± 2.70
61 years old and above	530 (12.7%)	67.04 ± 20.63	73.96 ± 8.45	7.63 ± 2.66
		*P*<0.001	*P* <0.001	*P* <0.001
Marital status				
Married/Cohabiting	3829 (91.2%)	71.77 ± 20.90	75.00 ± 8.18	7.14 ± 2.59
Single/widowed/divorced	367 (8.8%)	70.54 ± 21.03	72.47 ± 9.37	6.92 ± 2.59
		*P*>0.05	*P* = 0.005	*P* <0.001
Household income				
Very poor	611 (14.6%)	65.57 ± 22.67	74.08 ± 8.13	5.80 ± 2.77
Relatively poor	666 (15.9%)	69.31 ± 23.32	74.12 ± 8.68	6.23 ± 2.75
Moderate rich	980 (23.4%)	72.87 ± 21.73	75.11 ± 8.41	7.02 ± 2.57
Relatively rich	996(23.8%)	73.85 ± 19.61	74.63 ± 8.12	7.64 ± 2.26
Very rich	934(22.3%)	73.73 ± 17.70	75.50 ± 8.19	8.16 ± 2.09
		*P* <0.001	*P* = 0.004	*P* <0.001
Family educational level				
Junior middle school and below	1349 (32.2%)	69.24 ± 22.89	74.51 ± 8.31	6.22 ± 2.86
High school	1367 (32.6%)	72.23 ± 20.68	74.84 ± 8.54	7.20 ± 2.47
College school	754 (18.0%)	73.88 ± 19.17	74.90 ± 8.31	7.76 ± 2.21
Undergraduate and above	717 (17.2%)	72.81 ± 18.70	75.00 ± 7.91	7.98 ± 2.13
		*P* <0.001	*P* = 0.653	*P* <0.001
Family occupational status				
Peasant class	1978 (47.2%)	69.84 ± 22.09	75.00 ± 8.24	6.46 ± 2.76
Petty bourgeoisie class	556 (13.3%)	73.62 ± 21.67	74.59 ± 8.70	7.09 ± 2.45
Professional class	1185 (28.3%)	73.27 ± 19.28	74.34 ± 8.18	7.80 ± 2.12
Upper class	468 (11.2%)	73.00 ± 20.92	75.12 ± 8.52	8.20 ± 2.30
		*P* <0.001	*P* = 0.174	P <0.001
Family SES#				
Low	1583 (37.8%)	68.74 ± 22.81	74.67 ± 8.31	6.13 ± 2.79
Middle	1304 (31.1%)	73.36 ± 20.85	74.81 ± 8.65	7.31 ± 2.39
High	1300 (31.1%)	73.51 ± 17.99	74.86 ± 8.01	8.13 ± 2.06
		*P* <0.001	*P* = 0.808	*P* <0.001

#: Family SES was determined based on household income, educational level, and occupational status by using principle component analysis

### Family SES

Family SES in this study was quantified as a combination of household income condition, family educational level, and family occupation status. The household income condition was measured using the annual per capita household income, which was scored as follows: very poor (≤3000 RMB) = 1, relatively poor (3001–5000 RMB) = 2, moderately rich (5001–10,000 RMB) = 3, relatively rich (10,001–20,000 RMB) = 4, and very rich (> 20,000 RMB) = 5. Family education was categorized into four levels: junior middle school and below = 1, high school (or vocational school) = 2, college = 3, and undergraduate or above = 4. In accordance with the occupational classification found in previous studies [26, 27], occupations were rated as upper class (including higher-grade professionals, administrators, and officials) = 4, professional class (including lower-grade professionals, administrators and officials, and technicians) = 3, petty bourgeoisie class (including routine non-manual employees, service workers, sales personnel, and small proprietors) = 2, and peasant class (including manual workers, semi- and non-skilled manual workers, and agricultural workers) = 1. All family members’ educational level and occupational status were collected, and the highest levels for both were chosen to represent the family educational level and occupational status, respectively [28].

### Family social capital

In this study, family social capital was measured by using family cohesion and family support. Family cohesion was assessed based on the 16 items of cohesion subscale belonging to the Family Adaptability and Cohesion Evaluation Scale II (FACES II) [29]. This scale has high levels of reliability (internal consistency and test–retest) and validity (content and construct) [30]. All items were graded based on the following options: never = 1, rarely = 2, sometimes = 3, often = 4, and always = 5. Higher scores indicate better family relationship. The internal consistency reliability (Cronbach’s alpha) for FACES II in the current sample was 0.82. In this study, we focused on health-related family support. This was evaluated by asking questions such as the frequency with which family members exercised together, reminded each other to eat healthy, and reminded each other to have regular physical examinations. All items were scored based on the following options: always = 4, often = 3, sometimes = 2, hardly = 1, never = 0, and were together added as the total health-related family support score. The internal consistency reliability (Cronbach’s alpha) was acceptable at 0.72.

### Health outcome

The Health Survey Short Form (SF-36) was applied to evaluate health outcomes. Five general health perception items were used to measure the participants’ general health. The items were rated on a five-point Likert scale, and each perception item was combined to calculate the score (ranging from 0 to 100) based on the following scoring algorithm [31]:

$$ \mathrm{GH}=\frac{actual\ score-5}{20}\times 100, $$

Where *GH* indicates general health. The internal consistency reliability (Cronbach’s alpha) for the SF-36 in our study was acceptably high at 0.85.

### Covariates

Individual demographic variables, including gender, age, and marital status were used as covariates in the analyses. Gender was coded as female = 0 and male = 1. Marital status was a binary variable, with 0 denoting single/widowed/divorced and 1 denoting married/cohabiting.

### Statistical analysis

Population mean ± standard deviation for continuous variables (family cohesion, family health-related support, and general health) and proportions of categorical variables (gender, age group, marital status, household income condition, family education level, and family occupational status) were calculated. Using the principle component analysis, family SES was determined based on family household income, educational level, and occupational status. Family SES was further divided into low, middle, and high levels by using the quantile method. To test the relationships among family SES, family social capital, and family general health, the correlations among the variables were estimated. Further, a structural equation modeling analysis was conducted by using Mplus 7.0. Three models were tested, including a measurement model for family SES, a mediation model, and a full model. The structural equation model was estimated by using the maximum likelihood. Three goodness-of-fit indices were used for evaluating of the model fit—1) comparative fit index (CFI), whose value above 0.90 is considered to have a reasonable model fit [32]; 2) Tucker–Lewis index (TLI), like CFI, whose value, like CFI, should be close to 1 to indicate good model fit; and 3) root mean square error of approximation (RMSEA), whose value of 0.08 or below is regarded as a reasonable model fit.

The positive interaction term of “gender × health-related family support” demonstrates the effect of family health-related support on health differed by gender (Additional file Table 2). To obtain the standardized coefficients for model comparison, linear regression models were performed separately based on gender. SPSS 24.0 was used to generate the scale reliability coefficients, descriptive statistics, group comparisons, principle component analysis, variable correlation analysis, and linear regression model.

**Table 2:** Correlations between major variables

Variables	2	3	4	5	6
1. Household income	0.39***	0.44***	0.05***	0.32***	0.13***
2. Family educational level		0.53***	0.02	0.25***	0.07***
3. Family occupational status			0.02	0.26***	0.07***
4. Family cohesion				0.27***	0.23***
5. Health related family support					0.15***
6. General health					

*** *P* <0.001

## Results

Table 1 shows the demographic characteristics of participants as well as different population groups’ general health, family cohesion, and health-related family support score. A total of 4187 households were observed, the average age was 46.20 ± 11.85 years, 62.4% of the participants were female, and 91.2% were married or cohabiting. The mean score for general health was 71.66 ± 20.92. Participants who were male, younger, and with higher family SES reported a higher general health score. In comparison with men, women reported lower general health but higher health-related family support.

Results of correlation analysis of the major variables are presented in Table 2. Any two major variables showed statistically significant positive relationship (*r* range = 0.05–0.53), except the relationship between family educational level and family cohesion. The relationship between family occupational status and family cohesion appeared to be uncorrelated with each other.

### Relationships among family SES, family social capital, and general health

**Fig. 2:**
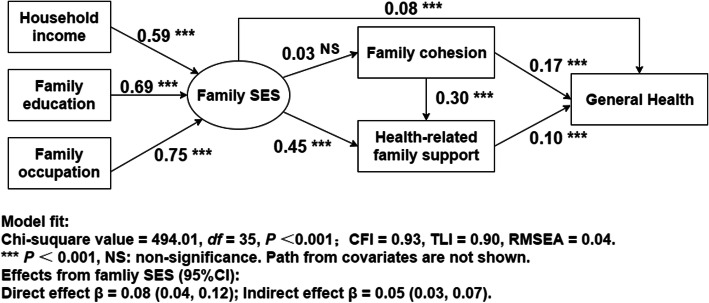
Standardized path coefficients for full model (*N* = 4187)

Figure 2 presents the specification for the final model. The indicators show a reasonable model fit with CFI of 0.93, TLI of 0.90, and RMSEA of 0.04. In terms of path coefficients, family SES had significant positive correlations with general health (β = 0.08, *P* < 0.001). In addition, family SES had an indirect effect on general health via family health-related support; the estimated mediation effect was 0.05 (0.03, 0.07). However, the coefficient for the indirect effect of family SES on general health via family cohesion was non-significant. Thus, the total effect of family SES on health was 0.13 (*P* < 0.001). These results support the formulation that family social capital serves as a mediator between family SES and adult general health, and that this mediating effect was mainly brought out through health-related family support.

Furthermore, family social capital, namely, family cohesion and health-related family support, was positively associated with individual general health, and the coefficients were 0.17 (0.16, 0.18) and 0.10 (0.08, 0.13), respectively. The effect of family social capital, that is, the combined effects of family cohesion and family health-related support was 0.27(β = 0.27, *P* < 0.001), which was greater than the total effect of family SES (β = 0.13, *P* < 0.001). The results also show family cohesion to be related to health-related family support in a predicted direction (β = 0.30, *P* < 0.001).

### Gender differences in the relationship between family social capital and general health

Table 3 shows the standardized coefficient of general health for men and women separately. By adjusting age, marital status, and family SES, family cohesion was found to be positively associated with general health for both men (β = 0.20, *P* < 0.001) and women (β = 0.16, *P* < 0.001). However, the association between health-related family support and health was greater for women (β = 0.13, *P* < 0.001) than for men (β = 0.04, *P* > 0.05).

**Table 3:** The standardized coefficient from regression model of general health for men and women separately

	Men	Women
Age	−0.19***	−0.21***
Married/Cohabiting	0.06*	0.04
Family SES	0.09***	0.09***
Family cohesion	0.20***	0.16***
Health-related family support	0.04	0.13***

***P <0.001, **P <0.01, *P <0.05

## Discussion

By using Chinese representative household data, this study attempted to understand the relationships among family SES, family social capital, and adult general health. It also attempted to test whether there are gender-based differences in this relationship. The results showed that both family SES and family social capital have indirect effects on adult general health. The total effect of family social capital on adult general health, including the effects of family cohesion and family health-related support, was greater than that of family SES. A positive relationship was also found between family cohesion and health-related family support. Furthermore, the results found women to more likely benefit from health-related family support than men.

As some evidence links social capital to health at the individual level, this study attempted to investigate this relationship at the family level. Family social capital was measured by utilizing family cohesion and health-related family support. The results show that family SES, family cohesion, and health-related family support are positively associated with adults’ general health, and the effect of family social capital on health is greater than that of family SES. Similar evidence was found concerning the children’s population. Both cross-sectional and longitudinal data showed family social capital to have a greater positive impact on children’s health than a family’s economic conditions [33, 34]. This study adds evidence to the formulation that family social capital plays an important role in adults’ health. In addition, health-related family support was found to play a partial mediating role between family SES and adult general health. The mediating effect of social support on individual SES and health outcome was already found in previous studies [35], and our study found a similar mediating effect in the family context. Families with higher SES have both economic capacity and health awareness to offer support to family members [36, 37]. Moreover, health-related family communication and support could influence family members’ understanding of health, improve their self-management ability [38, 39], reduce the burdens associated with stressful circumstances such as health care visits, and encourage healthy behavior adaptation [18]. Thus, increasing health-related family communication and support can help family members adopt healthy behaviors and promote their health.

This study found a positive relationship between family cohesion and health-related family support. As previous studies have already found better family cohesion to be associated with healthy and positive interactions among family members [40], this relationship was reconfirmed by our study. As a family is a dynamic system, families with appropriate cohesion tends to have good communication among its members, and thus effective family communication facilitates family cohesion [41]. This dynamic phenomenon might exist between family cohesion and health-related family support. Given the cross-sectional design, we only analyzed a one-way relationship between family cohesion and health-related family support in the current study.

This study also found gender-based differences in the relationship between family social capital and health. The correlation coefficient of health-related family support on general health was greater for women than men. This finding is consistent with the findings of previous studies [42, 43]. However, a study concerning Chinese elderly population found the associations between family social capital and life satisfaction to be higher among older men than women [44]. The difference between these results might be a because of the differences in ages between the subjects. In the relationship between social capital and health, both age- and gender-specific differences were found. With respect to middle age, the association between social support and health was found only for women; with respect to older age, the association was greater for men than women [45]. This might be partially because older women tend to have more social activities than older men, especially after retirement [45, 46]. Nearly 90% of the participants in this study were below the age of 60; thus, it is reasonable to state that the results are consistent with the middle-aged group than with the older group.

The limitations of our study should also be mentioned. First, social capital is relevant to the area of socio-cultural background [47]. While the findings of this study could be applicable in the Chinese context, one should be cautious when applying such evidence to another cultural context. Second, the current study adopts a cross-sectional design. Although it examined the associations among family SES, family social capital, and general health, it failed to identify the causality of these associations. Therefore, a prospective study is needed to understand the causal relationships among the components. Third, the measurement of family social capital used in this study needs further improvement. On the one hand, data on family social capital were collected from only one family member; the ideal way would have been to measure the social capital data of all family members and analyze them at the family member level or aggregate to family level [48]. On the other hand, this study only focused on health-related family support; a more comprehensive way would have been to assess family support that included emotional, instrumental, appraisal, and informational supports [49], which we recommend for future studies on this subject area.

## Conclusions

This study provides strong evidence for the positive associations between family SES, family social capital and adult health. First, health-related family support plays a mediating role between family SES and health, implying an atmosphere of good health-related family communication and support should also be emphasized, particularly for families with low SES. Meanwhile, health-related family support was associated with family cohesion, suggesting that family intervention programs, especially those targeting families with low levels of family cohesion, should focus on establishing a harmonious family relationship to mobilize family support. Emphasis on conflict resolution training to develop communication and problem-solving skills for family members [50] and encouragement of family members’ participation in interactive activities are both helpful to improve family cohesion [51]. Additionally, the gender-based differences imply that improving health-related family support could also be a health promotion strategy for women. There is a need for future studies that can focus on the specific forms of family support that effectively promote men’s health needs.

## Supplementary information

**Additional file 1.**

## Data Availability

The datasets used and analyzed during the current study available from the corresponding author on reasonable request.

## Abbreviations

SES: Socioeconomic status
S: Standard deviation
DF: Design effect
FACES II: Family adaptability and cohesion evaluation scale II
GH: General health
CFI: Comparative fit index
TLI: Tucker Lewis Index
RMSEA: Root Mean Square Error of Approximation
