# Supplementary material for: Family SES, family social capital, and general health in Chinese adults: exploring their relationships and the gender-based differences
Source: BMC Public Health. 2020 Sep 14;20:1401. doi: 10.1186/s12889-020-09508-5 (PMC7491135; doi:10.1186/s12889-020-09508-5)
Supplement: Supplementary file 1 — Additional file 1. [file 12889_2020_9508_MOESM1_ESM.docx]

Additional files

| Table 1. Sampling Methods (2014). | | | |
| --- | --- | --- | --- |
| Stage | Sampling unit | | |
|  | Eastern region | Central region | Western region |
| 1 | Two province-level regions were sampled from eastern regions. | Two province-level regions were sampled from central regions. | Two province-level regions were sampled from western regions. |
| 2 | In a selected province-level region, 4 county-level regions were sampled. | | |
| 3 | In a selected county-level region, 3 township-level regions were sampled. | | |
| 4 | In a selected township-level region, 2 committees were sampled. | | |
| 5 | In a selected committee, 30 households were sampled. | | |

| Table 2.Hierarchical regression of general health on demographic variables, family SES and family social capital. | | | | |
| --- | --- | --- | --- | --- |
|  | Standardized regression coefficients | | | |
|  | Step 1 | Step 2 | Step 3 | Step 4 |
| Gender | -0.09*** | -0.10*** | -0.08 | -0.22*** |
| Age | -0.20*** | -0.19*** | -0.19*** | -0.19*** |
| Marital status | 0.05*** | 0.04** | 0.04** | 0.04** |
| Family SES |  | 0.08*** | 0.08*** | 0.07*** |
| Family cohesion |  | 0.18*** | 0.19*** | 0.18*** |
| Health-related family support |  | 0.10*** | 0.10*** | -0.04 |
| Gender × family cohesion |  |  | -0.02 |  |
| Gender × health-related family support | |  |  | 0.19** |
| ***P ＜0.001, **P ＜0.01, *P ＜0.05 | | | | |
